# Production, characterization, and antifungal activity of a biosurfactant produced by *Rhodotorula babjevae* YS3

**DOI:** 10.1186/s12934-017-0711-z

**Published:** 2017-05-30

**Authors:** Suparna Sen, Siddhartha Narayan Borah, Arijit Bora, Suresh Deka

**Affiliations:** 1 Environmental Biotechnology Laboratory, Life Sciences Division, Institute of Advanced Study in Science and Technology(IASST), Vigyan Path, Paschim Boragaon, Garchuk, Guwahati, Assam 781035 India; 2 Department of Bioengineering and Technology, Institute of Science and Technology, Gauhati University, Jalukbari, Guwahati, Assam India

**Keywords:** Biosurfactant, *Rhodotorula babjevae*, Sophorolipid, Antifungal activity, LC–MS

## Abstract

**Background:**

Sophorolipids are one of the most promising glycolipid biosurfactants and have been successfully employed in bioremediation and various other industrial sectors. They have also been described to exhibit antimicrobial activity against different bacterial species. Nevertheless, previous literature pertaining to the antifungal activity of sophorolipids are limited indicating the need for further research to explore novel strains with wide antimicrobial activity. A novel yeast strain, *Rhodotorula babjevae* YS3, was recently isolated from an agricultural field in Assam, Northeast India. This study was primarily emphasized at the characterization and subsequent evaluation of antifungal activity of the sophorolipid biosurfactant produced by *R. babjevae* YS3.

**Results:**

The growth kinetics and biosurfactant production by *R. babjevae* YS3 was evaluated by cultivation in Bushnell-Haas medium containing glucose (10% w/v) as the sole carbon source. A reduction in the surface tension of the culture medium from 70 to 32.6 mN/m was observed after 24 h. The yield of crude biosurfactant was recorded to be 19.0 g/l which might further increase after optimization of the growth parameters. The biosurfactant was characterized to be a heterogeneous sophorolipid (SL) with both lactonic and acidic forms after TLC, FTIR and LC–MS analyses. The SL exhibited excellent oil spreading and emulsifying activity against crude oil at 38.46 mm^2^ and 100% respectively. The CMC was observed to be 130 mg/l. The stability of the SL was evaluated over a wide range of pH (2–10), salinity (2–10% NaCl) and temperature (at 120 °C for time intervals of 30 up to 120 min). The SL was found to retain surface-active properties under the extreme conditions. Additionally, the SL exhibited promising antifungal activity against a considerably broad group of pathogenic fungi viz. *Colletotrichum gloeosporioides*, *Fusarium verticilliodes*, *Fusarium oxysporum* f. sp. *pisi*, *Corynespora cassiicola*, and *Trichophyton rubrum*.

**Conclusions:**

The study reports, for the first time, the biosurfactant producing ability of *R. babjevae,* a relatively lesser studied yeast. The persistent surface active properties of the sophorolipid in extreme conditions advocates its applicability in diverse environmental and industrial sectors. Further, antifungal activities against plant and human pathogens opens up possibilities for development of efficient and eco-friendly antifungal agents with agricultural and biomedical applications.

## Background

Biosurfactants are widely known surface active agents of microbial origin produced by bacteria, yeasts or fungi. They are amphiphilic compounds with a hydrophobic and a hydrophilic moiety that tend to interact with phase boundaries in a heterogeneous system to solubilize the organic molecules [1]. These compounds comprise a wide range of chemical structures, such as glycolipids, lipopeptides, polysaccharide-protein complexes, phospholipids, fatty acids and neutral lipids [2, 3]. The environmentally hazardous production processes and by-products of chemical surfactants have effectively resulted in an increased interest in biosurfactants as possible alternatives [2]. They play an important role in various fields like bioremediation, biodegradation, oil recovery, food, pharmaceutics, and many other applications in different industrial sectors [3, 4]. Biosurfactants have carved a niche for themselves due to their unique environment-friendly properties and various benefits over their chemical counterparts such as low toxicity, higher biodegradability, high specificity, functionality under extreme conditions, and their possible production from different renewable sources [5].

Glycolipid biosurfactants are the most well studied microbial surfactant. The best-known glycolipid biosurfactants are rhamnolipids, trehalolipids, sophorolipids and mannosylerythritol lipids (MELs), which contain mono- or disaccharides, combined with long-chain aliphatic acids or hydroxy aliphatic acids [3]. Sophorolipids and mannosylerythritol lipids are representative glycolipid biosurfactants abundantly produced by various yeast strains [6]. Sophorolipids are produced mainly by yeasts, such as *Candida bombicola* (also known as *Torulopsis bombicola*), *Candida apicola* and *Rhodotorula bogoriensis*, while MELs are produced mainly by *Pseudozyma aphidis*, *Pseudozyma antarctica* and *Pseudozyma rugulosa* [7, 8]. SLs are composed of a hydrophobic fatty acid tail and a hydrophilic carbohydrate head composed of a disaccharide sophorose linked by a β-1, 2 bond which is optionally acetylated on the 6′ and/or 6′′ position. The structure of SLs is dependent on a terminal or sub-terminal hydroxylated fatty acid, which is linked β-glycosidically to the sophorose. The fatty acids’ carboxylic end can be free, forming the acidic structure or can be esterified at the 4′′ position giving rise to the lactonic ring structure [9]. Sophorolipids (SLs) are synthesized by non-pathogenic yeasts in contrast to another well-studied glycolipid, rhamnolipids (RLs), where the most efficient producer is the opportunistic pathogen *Pseudomonas aeruginosa*. Also, the production of SLs has been reported to be much higher as compared RLs leading to its wider commercialization. SLs have been documented to have a wide range of antimicrobial activity against several pathogens, the possible mechanism of which could be via membrane destabilization and increased permeabilization [10]. However, the antimicrobial activity has been described mostly against bacteria and in some cases against yeasts [11, 12]. Previous literature pertaining to the antifungal activity of sophorolipids are limited indicating the need for further research to explore novel strains with high productivity and wide applicability.

The present study was, therefore, undertaken to study the production, chemical structure (using FTIR and LC–MS), different physicochemical properties (surface tension reduction, emulsification index, critical micelle concentration, and stability studies), and antifungal activity of the sophorolipid biosurfactant produced by a locally isolated novel yeast *Rhodotorula babjevae* YS3.

## Methods

### Isolation of the yeast

For isolation of the yeast species, soil samples were collected in sterile plastic bags from a field located in Pathsala, Barpeta, Assam, India (26.4994°N, 91.1793°E). Isolation was carried out according to the methodology previously described [13]. Collected soil samples were placed in 50 ml plastic tubes, suspended in sterile water at concentrations of 5, 10, and 20% (w/v). Then, the soil suspensions were shaken in an orbital shaker at 200 rpm for 1 h. An aliquot of 0.15 ml was plated on yeast extract–peptone–dextrose (YPD) agar for cultivation experiments. Plates were then incubated at 19 ± 2 °C and examined after 7, 14 and 21 days of incubation. The observations were continued up to 21 days to provide sufficient time for the slow growing yeasts. Representatives of each morphologically distinct colony type were transferred into pure culture. Screening for biosurfactant producing isolates was performed based on the surface tension (ST) reduction ability of the growth medium (Bushnell Hass medium with 2% glucose as carbon substrate) measured using a digital tensiometer (Kruss K11, Germany), oil displacement tests [14], drop collapse test [15] and emulsification test [16].

### Gene sequencing and phylogenetic analysis of the yeast

The genomic DNA of the selected isolate YS3 was extracted using a kit purchased from Thermo Fisher Scientific (Cat No: 7870) according to manufacturer’s protocol. Regions of the rDNA containing the ITS and D1/D2 LSU domains were amplified using different combinations of the primers ITS1-F (CTTGGTCATTTAGAGGAAGTAA) [17], ITS1 (TCCGTAGGTGAACCTGCGG) [18], ITS4 (TCCTCCGCTTATTGATATGC) [18], and TW14 (GCTATCCTGAGGGAAACTT) [19]. The primer pair ITS1–ITS4 was used to amplify the ITS1-5.8S-ITS2 regions, while the primer pair of ITS1-F and TW14 was used to produce an amplicon containing the entire ITS and D1–D2 region [20]. The PCR product generated was purified with QIAquick PCR purification kit (Qiagen, Germany) for sequencing and later sequenced using an automated sequencer Beckman Coulter (GenomeLab GeXP, Genetic Analysis System, USA). The obtained sequence of the gene was analyzed using BLAST of the National Center for Biotechnology Information. Related sequences were aligned using ClustalW software [21] and the phylogenetic tree was visualized using Mega 7 software [22].

### Preservation and inoculum preparation of the culture

A pure culture of YS3 was maintained in 30% (v/v) glycerol and stored at −80 °C. For routine experiments, the strain was maintained at 4 °C in YPD agar slants containing (in g/l): yeast extract 10, peptone 20, and dextrose 20. Transfers were made to fresh agar slants each month to maintain viability. For active culture preparation, scrapings from the slants were added to 100 ml YPD broth in 250 ml Erlenmeyer flasks and incubated at 19 °C in a rotary shaker at 200 rpm for 72 h. For seed culture preparation, 5% inoculum was transferred to a flask with 100 ml Bushnell-Hass medium (BHM) with 10% glucose (w/v) and incubated under the same set of growth conditions.

### Biomass determination and biosurfactant extraction

For biomass determination, 100 ml of the culture broth was taken in pre-weighed tubes and centrifuged at 12,000*g* for 20 min. The pellet was washed twice with distilled water and dried in a hot air oven at 105 °C for 24 h [23]. The dry weight of the cells was then determined.

Biosurfactant extraction was carried out according to Luna et al. [24] with some modifications. The cell-free culture broth was acidified with 6 N HCl to pH 2.0 and left overnight at 4 °C. The culture broth was then extracted with an equal volume of ethyl acetate thrice, and the organic phase was vacuum-dried at 40 °C to remove the solvent. Biosurfactant yield and biomass were expressed in g/l.

### Purification of the biosurfactant

Purification of the biosurfactant by silica gel column chromatography was carried out according to Daverey and Pakshirajan [25] with some modifications. A 26  ×  3.3 cm^2^ glass column was packed with a slurry containing silica gel 60–120 mesh in chloroform. The column was loaded with 1 g of crude biosurfactant dissolved in 5 ml chloroform and elution was carried out with chloroform: methanol using gradient system (0–50% methanol). Fractions obtained were pooled and vacuum dried at 40 °C.

### Physico-chemical characterization of the biosurfactant

#### Surface tension (ST) measurement and critical micelle concentration (CMC)

The fermentation broth was collected every 24 h for 240 h and ST was recorded with a tensiometer using the Wilhelmy plate method at 25 °C. The instrument was calibrated against ultrapure water (ST 72.8 mN/m) for accurate measurements. CMC was determined according to standard methods [26]. ST of the column purified biosurfactant at concentrations ranging from 10 to 200 mg/l was recorded until a constant value was reached. Finally, the first concentration at which the ST became constant was determined as the CMC.

#### Emulsification index

The emulsifying activity measured as the emulsification index was determined according to Cooper and Goldenberg [16]. Equal amounts of oil substrates (crude oil, sunflower oil, engine oil, n-hexadecane, and diesel) and the cell-free culture broth were taken in test tubes, vortexed at high speed for 2 min and left undisturbed. Emulsion stability after 24 and 168 h was calculated as Emulsification index (EI) = (Height of emulsion layer/Height of the total mixture) × 100.

#### Stability studies

The stability of the biosurfactant was tested in terms of temperature, pH, and salinity. To determine the thermal stability in terms of ST, the cell-free culture broth was heated for different time intervals (30–120 min) at 120 °C and ST was recorded after cooling the broth to room temperature. To study the effect of pH on stability, the cell-free broth was adjusted to desired values of pH (2–10) using either 6 N NaOH or 6 N HCl followed by the ST measurement. The effect of salinity (2–10% NaCl, w/v) on the activity of the biosurfactant was investigated in a similar manner [27].

### Compositional analyses of the biosurfactant

#### Thin layer chromatography (TLC)

The column purified biosurfactant was dissolved in methanol and spotted on silica gel plate (Merck DC, Silica gel 60 F_254_). The mobile phase was composed of chloroform: methanol: water (65: 15: 2, v/v). Once dry, the plate was developed in a chamber saturated with iodine fumes for detection of lipids and subsequently sprayed with anthrone reagent for sugar detection. A commercially available SL, 1,4′′-sophorolactone 6′,6′′-diacetate (Sigma-Aldrich, USA) was used as a reference standard.

#### Fourier Transform Infrared Spectroscopy (FTIR)

The FTIR spectra were recorded in a Nicolet 6700 FTIR System (Thermo Scientific, Waltham, MA, USA). FTIR of the test biosurfactant along with the standard SL, 1,4′′-sophorolactone 6′,6′′-diacetate in attenuated total reflectance (ATR) mode was performed at a resolution and wave number accuracy of 4 and 0.01 cm^−1^, respectively, and 32 scans with correlation for atmospheric CO_2_ [28]. All data were corrected for the background spectrum.

#### Liquid chromatography–mass spectrometry (LC–MS)

Separation of the biosurfactant and the standard sophorolactone along with identification of the different structural homologues were performed with LC–MS using an Agilent 1260 HPLC equipped with a C18 column (100, 2 mm i.d., 3 μm particle diameter) [29]. The mobile phases consisted of water spiked with 0.5% formic acid (v/v) (A) and acetonitrile (B). The mobile phase flow rate was 0.2 ml/min, and the following gradient was employed: 5% B ramped to 70% B in 3 min (linear) and then ramped to 80% B in 12 min (linear), followed by a linear increase to 95% B in 3 min (held for 8 min) and then a change to 5% in 1 min (held for 3 min). The HPLC was interfaced with a 6410 triple quadrupole mass spectrometer equipped with electrospray ionization (ESI). ESI–MS was performed in positive ion mode and analyzed using Agilent MassHunter software. Full scan data were obtained by scanning from m/z 50–1000 with a fragmentor voltage calibrated at 135.0 V.

#### Antifungal activity

The antifungal activity of the biosurfactant against certain plant and human pathogens viz. *Colletotrichum gloeosporioides* (ITCC 6434), *Fusarium verticilliodes* (MTCC 10556), *Fusarium oxysporum* f. sp. *pisi* (ITCC 4814), *Corynespora cassiicola* (ITCC 6748), and *Trichophyton rubrum* (MTCC 8477) was studied using microbroth dilution technique in a serial twofold dilution using a 96-well flat bottom microtiter plate [30]. Fungal inocula were prepared in Potato dextrose broth (PDB) except for *T. rubrum*, for which Sabouraud dextrose broth (SDB) was used. Then, each well was inoculated with 10 μl of spore suspension at a concentration of 1 × 10^7^ cfu/ml. Un-inoculated wells containing PDB or SDB with and without biosurfactant served as controls. The plates were incubated for 48 h at 25 °C. The absorbance of each well at 600 nm was measured by Multimode Reader (Thermo Scientific, Waltham, MA, USA) and MIC was described as the lowest concentration at which no growth was observed.

### Statistical analysis

All the experimental data were expressed in terms of arithmetic averages of triplicates, and the error bars indicates the standard error of mean (SEM). One sample t test was used to compare the differences in biosurfactant yield (*p* < 0.05) using the statistical package for the Social Sciences (IBM SPSS Statistics 22.0, IBM Corp. Armonk, USA).

## Results

### Isolation and identification of biosurfactant producing yeast

A total of fifteen morphologically distinct yeasts were isolated from soil samples as pure cultures (designated as YS1–YS15). Among the isolates, only one strain YS3 showed promising results for all the screening tests with an area of 38.46 mm^2^ in oil displacement test, positive activity in drop collapse, 100% emulsification index (E_24_) against crude oil, and ST reduction from 72.8 to 34.8 mN/m.

**Fig. 1:**
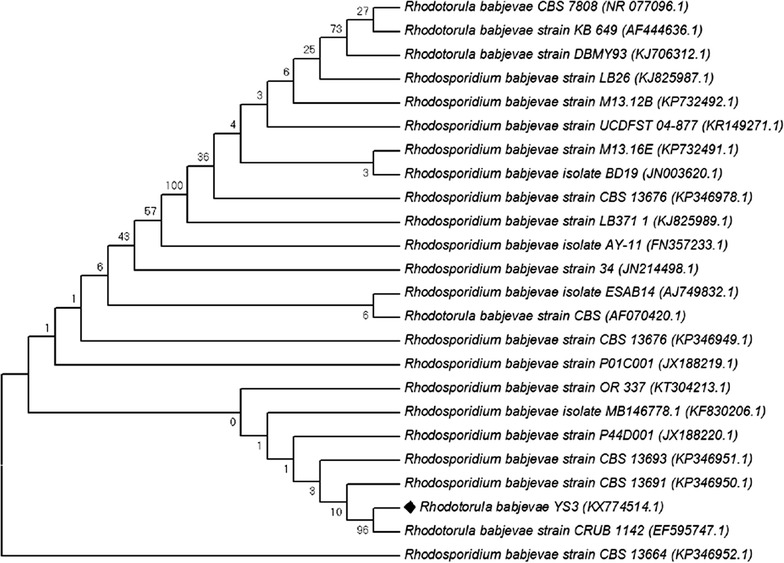
Phylogenetic tree of *Rhodotorula babjevae* YS3 and its related sequences retrieved from NCBI database

The sequencing of the amplicon for the ITS1-5.8S-ITS2 region generated a contig sequence of 429 bp. A BLAST search with available sequences in the NCBI database revealed 100% identity with *R. babjevae* strains LB371_1 and LB139.2, GenBank accessions KJ825989 and KJ825988 respectively [31]. Similarly, the longer sequence comprising of the ITS and D1-D2 region (1460 bp) showed 99% identity with *R. babjevae* strains P01C001 (JX188219) and P44D001 (JX188220) [20]. Both the sequences (429 and 1460 bp) were submitted to GenBank (accession Nos. KU600016 and KX774514 respectively). Based on these results, the yeast isolate YS3 was identified as *R. babjevae*. The evolutionary relationship of *R. babjevae* using neighbor-joining method has been depicted in Fig. 1.

### Growth kinetics and biosurfactant production

**Fig. 2:**
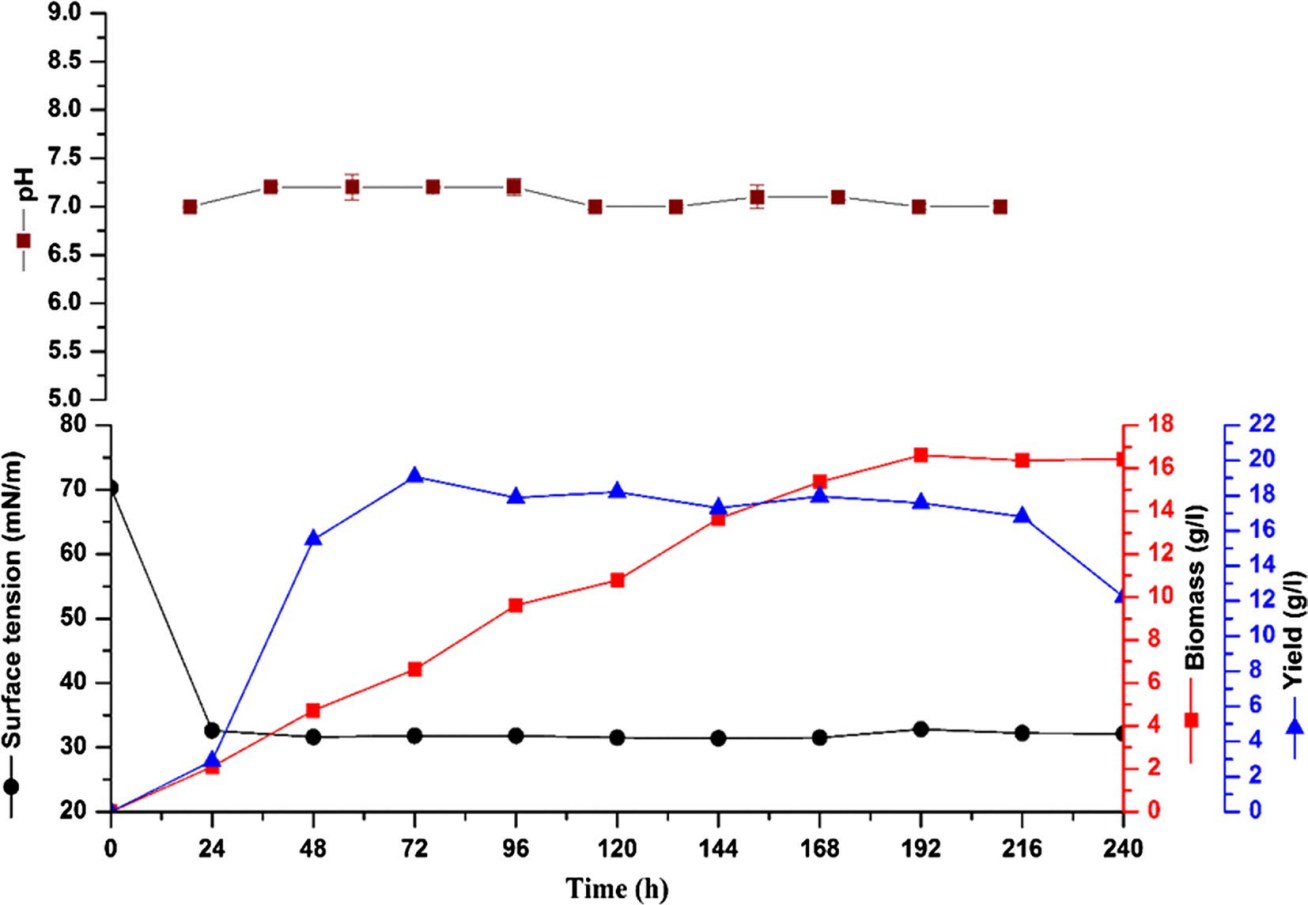
Growth kinetics, pH, surface tension, and biosurfactant yield of *Rhodotorula babjevae* YS3 grown at 19 °C, 200 rpm, 5% inoculum (v/v), 10% glucose (w/v) plotted as a function of time. *Error bars* illustrate standard error of mean (SEM), calculated from two independent experiments in triplicates

Biosurfactant production and the growth characteristics of *R. babjevae* at 19 °C are presented in Fig. 2. As evident from the results displayed, the growth of the yeast started without a lag time and the exponential phase prolonged till 168 h. During this period, the biomass value was recorded at 11 g/l. However, maximal cell biomass production (16.61 g/l) occurred during the stationary phase after 192 h of growth. The biosurfactant yield was the highest in the exponential growth phase at 72 h (19.0 g/l) dropping significantly after 216 h (12.23 g/l) [*t*(10) = 7.146, *p* = 0.000]. The pH of the uninoculated fermentation medium was 7.0 and did not vary significantly [*t*(10) = 2.206, p = 0.052] throughout the experiment. ST of the culture reduced from 70 to 32.6 mN/m after 24 h of cultivation during the early exponential phase and remained stable throughout the incubation period of 240 h.

### Surface tension (ST) measurement and critical micelle concentration (CMC)

**Fig. 3:**
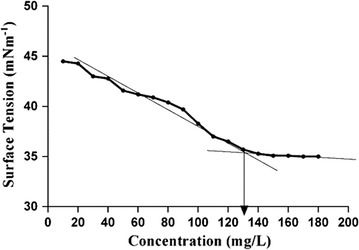
CMC and minimum surface tension of the biosurfactant produced by *Rhodotorula babjevae* YS3. *Arrow* depicts the CMC value

Critical to any surface active compounds are its properties such as ST and critical micelle concentration (CMC) [32]. The biosurfactant from *R. babjevae* reduced ST from 72.8 to 35 mN/m with an increase in the concentration of the biosurfactant up to 130 mg/l (Fig. 3), at which point, a further increase in biosurfactant concentration did not have an effect on the ST.

### Emulsification index (EI)

The emulsification activity of the biosurfactant and stability of the emulsion after 24 and 168 h was studied in different hydrophobic substrates as shown in Table 1. The biosurfactant could effectively emulsify and stabilize the emulsion formed with crude and motor oil, but EI (%) against crude oil was the highest with 98% stability of the emulsion after 168 h. However, the activity was weaker in n-hexadecane and diesel with retention of 88 and 20% activity respectively after the same duration.

**Table 1:** Emulsification index (EI) evaluated using the biosurfactant containing culture broth of *Rhodotorula babjevae* YS3 grown at 19 °C, 200 rpm, 5% inoculum (v/v), 10% glucose (w/v) after 24 and 168 h

Sl. no	Hydrophobic substrates	Emulsification index (%)	
		24 h	168 h
1	n-Hexadecane	25.19 ± 0.12	22.58 ± 0.11
2	Sunflower oil	33.33 ± 0.87	5.26 ± 0.53
3	Motor oil	62.26 ± 0.32	60.00 ± 0.12
4	Diesel	25.00 ± 0.25	5.00 ± 0.43
5	Crude oil	100.00 ± 0.32	83.33 ± 0.23

Values are mean ± SEM of triplicates with three independent experiments

### Stability studies

**Fig. 4:**
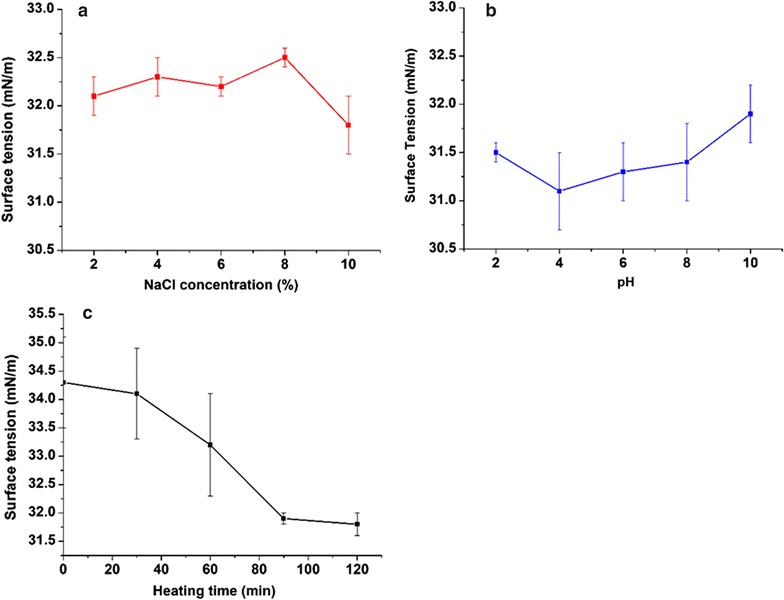
Effect of NaCl concentration (%) (**a**), pH (**b**), and heating time (min) (**c**) at 120 °C on ST of the culture supernatants of *Rhodotorula babjevae* YS3 grown at 19 °C, 200 rpm, 5% inoculum (v/v), 10% glucose (w/v).* Error bars* illustrate standard error of mean (SEM), calculated from two independent experiments in triplicates

The effect of salinity concentrations, heating time and pH on the stability of the biosurfactant was investigated in terms of ST (Fig. 4a–c). The ST reducing ability of the biosurfactant remained practically unaltered with increasing NaCl concentrations ranging from 2 to 10%. Similarly, studies on the effect of pH on the cell-free culture broth showed no significant alteration in the ST. The biosurfactant also showed excellent stability after heating at 120 °C for different time intervals.

### Compositional analyses of the biosurfactant

#### Thin layer chromatography (TLC)

**Fig. 5:**
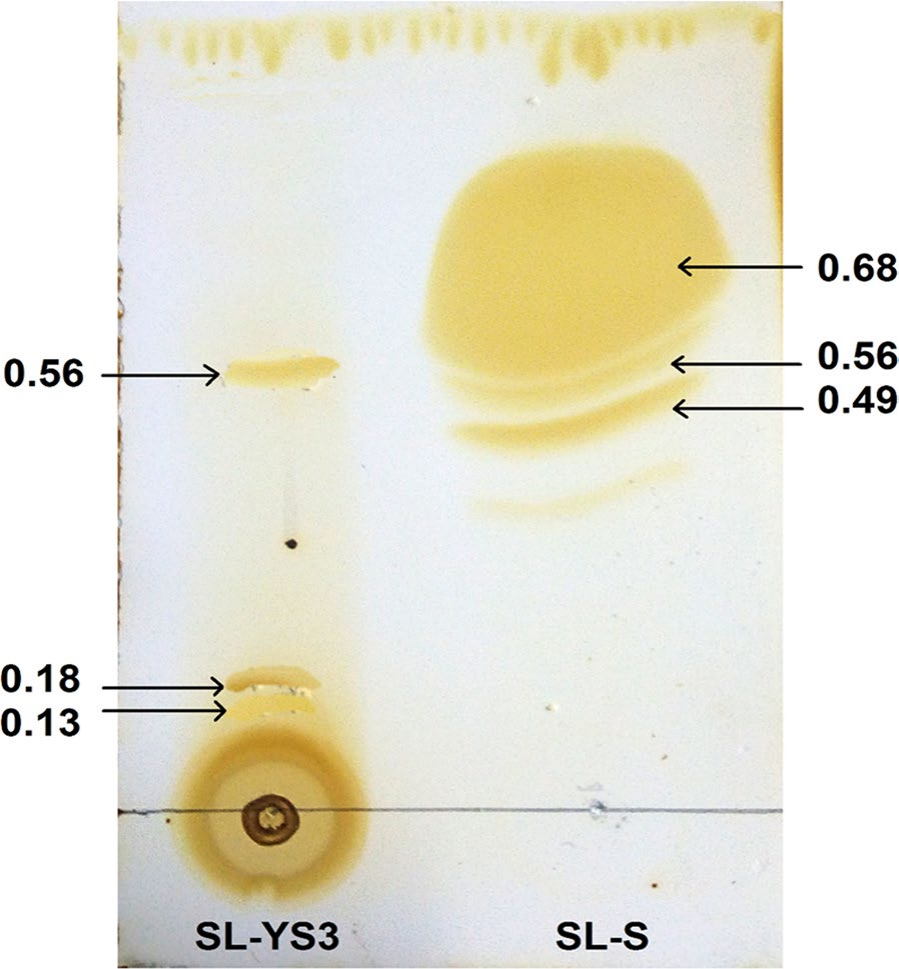
TLC chromatogram showing the separation of components of sophorolipid biosurfactant produced by *Rhodotorula babjevae* YS3 (SL-YS3) in comparison to the sophorolipid standard, 1,4′′-sophorolactone 6′,6′′-diacetate (SL-S)

Thin layer chromatography was used as a preliminary method for compositional analysis of the biosurfactant. A positive reaction to sugars and lipids using anthrone reagents and iodine vapours respectively confirmed the biosurfactant produced by *R. babjevae* YS3 to be a glycolipid. As revealed by the TLC (Fig. 5) chromatogram, the chemical nature of the biosurfactant was predicted as SL on comparison with the standard SL (1,4′′-sophorolactone 6′,6′′-diacetate) and with R_*f*_ values mentioned in literature [33–35]. The standard lactonic SL (LS) appeared as three spots with R_*f*_ values 0.49, 0.56 and 0.68. The spot with R_*f*_ value 0.56 was also observed in the test sample indicating the presence of LS. Apart from that, two spots corresponding to acidic SL (AS) were also detected in the biosurfactant with R_*f*_ values 0.13 and 0.18.

#### Fourier transform infrared spectroscopy (FTIR)

**Fig. 6:**
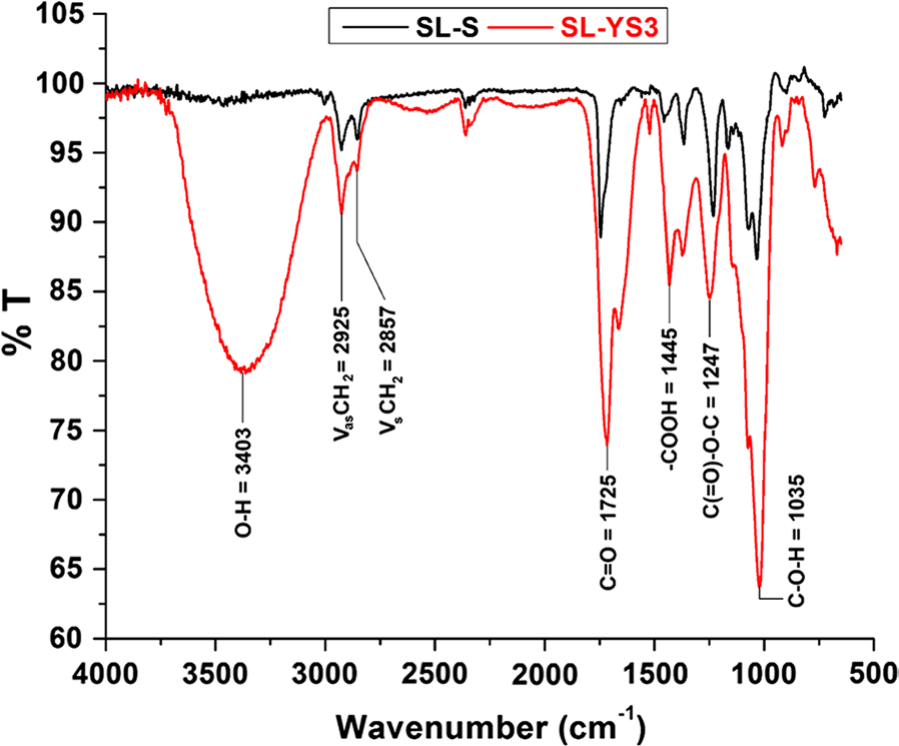
FTIR spectra of the biosurfactant produced by *Rhodotorula babjevae* YS3 (SL-YS3) and sophorolipid standard, 1,4′′-sophorolactone 6′,6′′-diacetate (SL-S)

The FTIR spectra of the test biosurfactant and the standard were fairly similar (Fig. 6). The asymmetrical stretching (ν_as_ CH_2_) and symmetrical stretching (ν_s_ CH_2_) of methylene groups occurred at 2925 and 2857 cm^−1^, respectively. Further, the absorption band at 1725 cm^−1^ corresponding to the presence of lactone (C=O) group was also detected. The stretch of C–O band of C(=O)–O–C in lactones was represented by a band at 1157 cm^−1^, whereas that from the acetyl esters was found at 1247 cm^−1^. Moreover, the sugar C–O stretch of C–O–H groups was found at 1035 cm^−1^. The primary difference observed in the spectra of the test biosurfactant, and the standard was the absence of a band at 3403 cm^−1^ (O–H stretch) in the standard as opposed to a broad band in the former. Also, a much stronger absorption band for C–O–H in-plane bending of carboxylic acid (–COOH) was observed at 1445 cm^−1^ in the test biosurfactant as compared to the standard. These two bands at 1445 and 3403 cm^−1^ were found to be typical with AS in literature [25] confirming that the test biosurfactant was a mixture of both LS and AS.

#### Liquid chromatography–mass spectrometry (LC–MS)

**Fig. 7:**
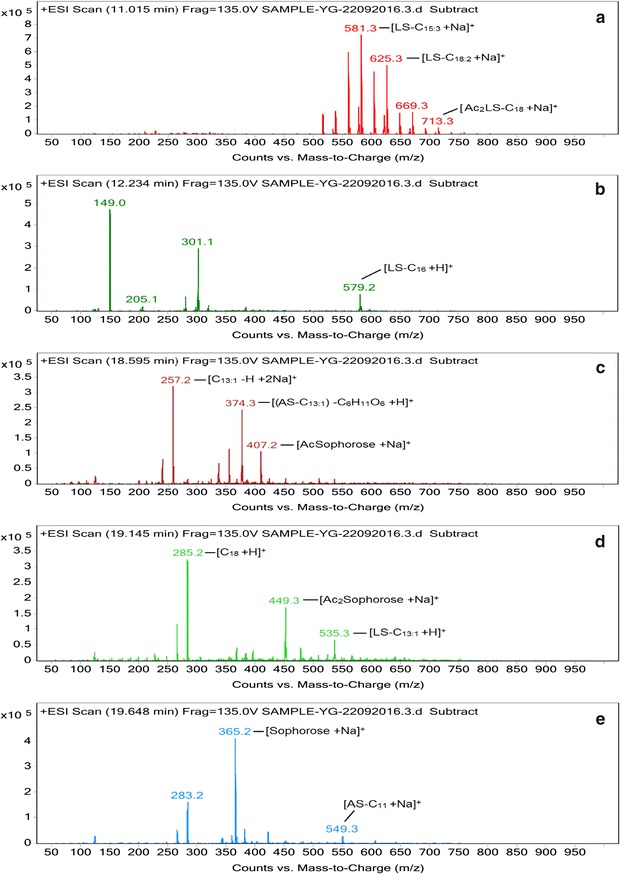
Characterization of the biosurfactant (YG) produced by *Rhodotorula babjevae* YS3 with glucose as the sole carbon source using LC–MS in positive electrospray ionization mode (+ESI). **a** MS showing the sodiated adducts of lactonic sophorolipids (LS) with pentadecatrienoic (C_15:3_) and octadecadienoic (C_18:2_) lipid side chains at m/z values 581 and 625 respectively, while the adduct ion at m/z 713 represents di-acetylated lactonic sophorolipid (Ac_2_LS) with octadecanoic (C_18_) lipid chain. **b** The ion at m/z 579 corresponds to lactonic sophorolipid with hexadecanoic lipid chain (LS-C_16_). **c** Disodiated adduct ion of the fragmented tridecenoic fatty acid side chain was observed at m/z 257, the protonated ion at m/z 374 represents the acidic sophorolipid with tridecenoic acidic chain (AS-C_13:1_) after loss of the terminal hexose (C_6_H_11_O_6_) from the sophorose disaccharide, sodiated adduct of fragmented mono-acetylated disaccharide moiety (AcSophorose) was observed at m/z 407. **d** The protonated ions at m/z 285 and 535 represent fragmented octadecanoic fatty acid side chain and lactonic sophorolipid with tridecenoic lipid chain (LS-C_13:1_) respectively; the sodiated adduct of fragmented di-acetylated sophorose moiety (Ac_2_Sophorose) was observed at m/z 449. **e** The sodiated adducts of the fragmented sophorose moiety and the acidic sophorolipid with undecanoic acidic side chain (AS-C_11_) were observed at m/z 365 and 549 respectively

The LC–ESI–MS of the test sample as well as the standard sophorolactone were acquired in the positive mode and exhibited both protonated as well as sodiated adduct ions. The mass spectral ions were identified by calculation of elemental composition and comparison with available literature [35, 36]. The purified biosurfactant sample was a mixture of five components. The MS chromatograms for each eluting fragment for the test sample are presented in Fig. 7a–e. The test sample contained a total of five LS and two AS homologues with lipid chains of varying lengths and unsaturation. LS-C_15:3_ was detected as [M+H]^+^ and [M+Na]^+^ at m/z 559 and 581 respectively, while the same ions for the co-eluting LS-C_18:2_ were detected at respective m/z values of 603 and 625. The peak at m/z 713 correspond to [M+Na]^+^ ion of the diacetylated (Ac_2_) Ac_2_LS-C_18_. The [M+H]^+^ ions for LS-C_16_ and LS-C_13:1_ were observed at m/z 579 and 535 respectively. The peak at m/z 549 corresponds to the [M+Na]^+^ ion of the acidic sophorolipid AS-C_11_. Apart from these intact sophorolipids, fragment ions were also observed in the MS chromatograms. The peak with retention time 18.595 min gave peaks at m/z 257, 374 and 407. The peak at 257 corresponds to the disodiated tridecenoic fatty acid chain fragment. The peak at 374 represents the protonated ion generated after the loss of terminal hexose (C_6_H_11_O_6_) from the AS containing a tridecenoic fatty acid moiety (AS C_13:1_), while the fragment at 407 represents sodiated adduct of the monoacetylated (Ac) disaccharide after the loss of the fatty acid moiety. The fragments at m/z 285 and 449 correspond to the protonated octadecanoic hydroxy fatty acid moiety and the sodiated diacetylated disaccharide fragments respectively. The fragment at 365 represents the sodiated adduct of the unacetylated disaccharide unit originated from the loss of the fatty acid moiety (186 Da) from AS-C_11_.

**Fig. 8:**
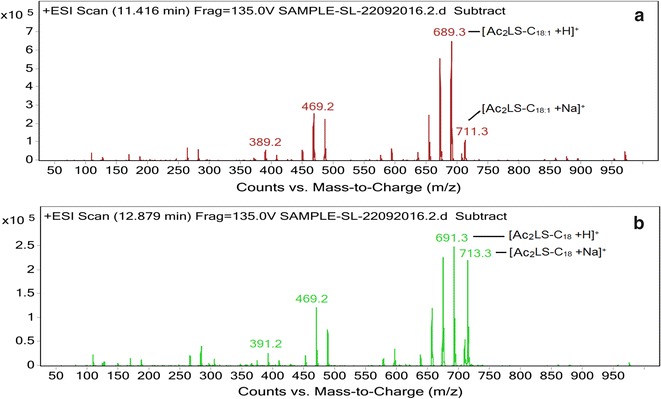
LC–MS spectra of the standard sophorolactone (SL), 1,4′′-sophorolactone 6′,6′′-diacetate in positive electrospray ionization mode (+ESI). **a** MS showing the protonated ion and sodiated adducts of di-acetylated lactonic sophorolipids (Ac_2_LS) with octadecenoic (C_18:1_) lipid side chains at m/z values 689 and 711 respectively. **b** The peaks at m/z 691 and 713 represent protonated ion and sodiated adduct of Ac_2_LS with octadecanoic (C_18_) lipid chain

**Fig. 9:**
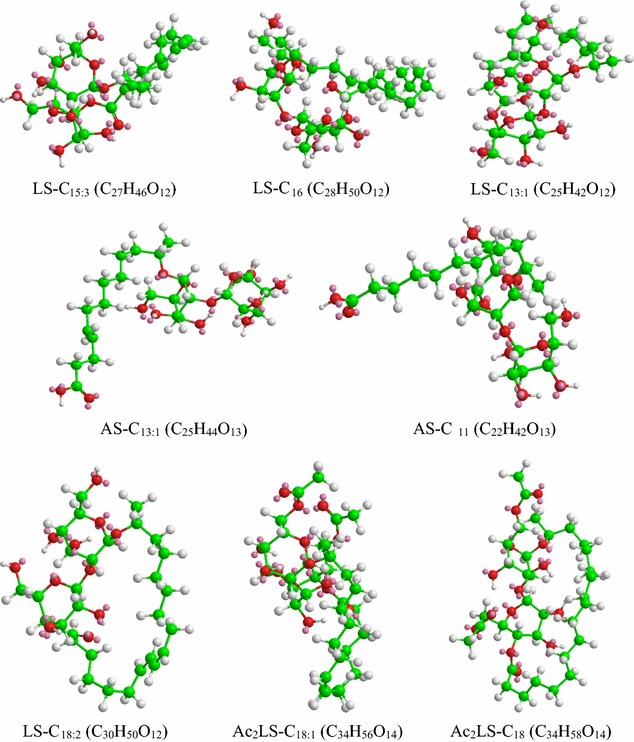
The least energy structures of the sophorolipid (SL) homologues detected during LC–MS analyses of the SL produced by *Rhodotorula babjevae* YS3 and SL standard, 1,4′′-sophorolactone 6′,6′′-diacetate. Structures were drawn in ChemDraw Ultra 12.0 (*LS* lactonic SL, *AS* acidic SL, *Ac* acetyl group)

The standard sophorolactone was found to contain two constituent sophorolipids under the same test conditions (Fig. 8). The corresponding MS spectra revealed the presence of both protonated as well as sodiated adduct ions of two Ac_2_ LS viz. Ac_2_LS-C_18:1_ and Ac_2_LS-C_18_. The peak with retention time 11.416 min revealed peaks at m/z 689 and 711 corresponding to the [M+H]^+^ and [M+Na]^+^ ions of the Ac_2_ sophorolactone with C_18:1_ monounsaturated fatty acid moiety (Fig. 8a). The second peak with retention time 12.879 min revealed peaks at m/z 691 and 713 corresponding to the [M+H]^+^ and [M+Na]^+^ ions of the Ac_2_ sophorolactone with C_18_ saturated fatty acid moiety (Fig. 8b). The complete list of sophorolipid homologues detected in both the test as well as the standard sample along with their chemical formula, molecular mass, type, and source are presented in Table 2 and their least energy structures are presented in Fig. 9.

**Table 2:** The chemical formula, molecular mass, and type of the sophorolipid (SL) homologues produced by *Rhodotorula babjevae* YS3 (SL-YS3), along with the sophorolipid standard, 1,4′′-sophorolactone 6′,6′′-diacetate (SL-S) as determined by LC–MS (*LS* lactonic SL, *AS* acidic SL, *Ac* acetyl group)

Homologue	Formula	Molecular mass	Type of SL	Source
AS-C_11_	C_22_H_42_O_13_	514.56	Acidic	SL-YS3
LS-C_13:1_	C_25_H_42_O_12_	534.59	Lactonic	SL-YS3
AS-C_13:1_	C_25_H_44_O_13_	552.61	Acidic	SL-YS3
LS-C_15:3_	C_27_H_46_O_12_	562.65	Lactonic	SL-YS3
LS-C_16_	C_28_H_50_O_12_	578.69	Lactonic	SL-YS3
LS-C_18:2_	C_30_H_50_O_12_	602.71	Lactonic	SL-YS3
Ac_2_LS-C_18:1_	C_34_H_56_O_14_	688.80	Lactonic	SL-S
Ac_2_LS-C_18_	C_34_H_58_O_14_	690.81	Lactonic	SL-YS3, SL-S

*LS* lactonic SL, *AS* acidic SL, *Ac* acetyl group. SL-YS3 = Sophorolipid by *Rhodotorula babjevae* YS3, SL-S = sophorolipid standard, 1,4′′-sophorolactone 6′,6′′-diacetate

#### Antifungal activity

The in vitro antifungal activities of the column purified SL and the standard against certain plant and human fungal pathogens were estimated on the basis of MIC values (Table 3) obtained in the range of 62–1000 µg/ml. The test SL exhibited promising antifungal activities against *C. gloeosporioides*, *F. oxysporum* f. sp. *pisi*, and *F. verticillioides* as evident from the low MIC values. However, no inhibitory activity was observed against *C. cassiicola* at the evaluated concentrations in this study.

**Table 3:** Antimicrobial activity of the sophorolipid produced by *Rhodotorula babjevae* YS3 (SL-YS3) in comparison to sophorolipid standard, 1,4′′-sophorolactone 6′,6′′-diacetate (SL-S) against pathogenic fungal strains

Sl. no.	Fungal strains	Minimum inhibitory concentration (MIC, µg/ml)	
		SL-YS3	SL-S
1	*Colletotrichum gloeosporioides*	62	50
2	*Fusarium verticilliodes*	125	125
3	*Fusarium oxysporum* f. sp. *pisi*	125	125
4	*Corynespora cassiicola*	>2000	1000
5	*Trichophyton rubrum*	1000	1000

SL-YS3 = Sophorolipid by *Rhodotorula babjevae* YS3, SL-S = sophorolipid standard, 1,4′′-sophorolactone 6′,6′′-diacetate. Values are mean ± SEM of triplicates with three independent experiments

## Discussion

This study reports the biosurfactant producing ability of a hitherto unreported yeast *R. babjevae* strain YS3 which was subsequently characterized as a sophorolipid (SL) with appreciable antifungal activity and stable surface-active properties. The yeast diversity in soil has not yet been explored in great detail and as such offers a huge scope for isolation of yeasts with several unique properties [13]. We screened for the biosurfactant producing ability of the pigmented yeast *R. babjevae* YS3 based on its oil displacement, drop-collapse, emulsification activity and surface tension reduction ability. The oil displacement technique is a biosurfactant detection method based on its surface activity and it is directly proportional to biosurfactant concentration in the medium [14, 15]. The drop collapse method determines qualitative examinations whether the broth has sufficient amount of biosurfactant to collapse or evenly spread its cell-free drops on the hydrophobic surface [37]. Emulsification activity and ST reduction are basic properties of biosurfactant [38]. Potential biosurfactant producing microbes are expected to reduce the ST of the culture media to nearly 35 mN/m [39]. Accordingly, the strain YS3 was able to reduce the ST below this value indicating successful biosurfactant production. Previously, biosurfactant producing yeast have reportedly been isolated from hydrocarbon contaminated soil [6], but the isolation of biosurfactant producing yeast from agricultural soil in our study further diversifies the potential sources for exploration of biosurfactant producing yeasts.

Depending upon the nature of the biosurfactant and the producing microorganisms, several patterns of biosurfactant production by fermentation are possible [40]. In our study, growth curves were obtained in order to establish the relation between cell growth, surface-activity, yield of the biosurfactant and pH in time. In our study, ST reduction and biosurfactant production was observed after 24 h when growth was almost non-existent. Significant increase in the production was observed at 48 h with an increase in cell growth although the ST did not vary significantly till the end of the cultivation period. The highest biosurfactant production was observed after 72 h during the exponential phase indicating a growth associated biosurfactant production. Similar growth associated production of biosurfactant was also obtained by Diniz Rufino et al. [41] when they cultivated *Candida lipolytica* in a medium supplemented with 6% soybean oil refinery residue and 1% glutamic acid. Accorsini et al. [42] reported that the highest reduction in ST due to biosurfactant production by *C. antartica* was observed after 24 h of growth from an initial value of 47–31 mN/m. However, some researchers have also reported biosurfactant production by yeasts during their stationary growth [43]. The pH was not controlled in this study and alteration in its value was non-significant throughout the cultivation period of 240 h. Similar result was reported by Luna et al. [44] and they inferred that the high biosurfactant yield as observed by them could be a result of the slight alteration in the pH throughout the experiment duration. Interestingly, in our study no secondary hydrophobic carbon source was added to the production media contrary to previous reports where the biosurfactant production by yeasts was achieved through the co-substrate fermentation of glucose and a fatty acid [24, 41, 43]. Nevertheless, the biosurfactant yield was higher in our study as compared to the ones mentioned in these reports, indicative of the fact that the secondary carbon source may not play a critical role in the production of biosurfactant in our case. This will help to make the production process more economical by avoiding the additional cost of a secondary source.

Critical micelle concentration (CMC) is an integral property of any surface active compound and plays a crucial role in its characterization [32]. CMC corresponds to the minimum concentration of surfactant at which the surfactant monomers start to form micelles, because at this point, the solution interface of the medium in which the surfactant is dispersed gets fully saturated with surfactant molecules [45]. Beyond the CMC, no significant lowering in ST is observed and as such a lower CMC value indicates a higher efficiency of a surfactant as lesser amount is required to lower the ST [46]. The CMC values obtained in our study are consistent with the findings of Otto et al. [42], where they reported a CMC and minimum ST value of 130 mg/l and 39 mN/m respectively for a mixture of sophorolipids produced in a two-stage process using *C. bombicola* grown on deproteinized whey and rapeseed oil as the carbon sources.

The SL sample under study could form stable emulsions with crude oil, motor oil and n-hexadecane. Although the emulsification index was relatively lower against n-hexadecane, the emulsion was stable over the evaluation period of 168 h. Emulsification activity of a surfactant involves dispersion of one liquid phase as microscopic droplets in another (continuous) resulting in the mixing of two immiscible liquid. This characteristic is a crucial factor for their utilization in various industrial sectors [47]. Most microbial surfactants are substrate specific and solubilize or emulsify different hydrocarbons at different rates depending on its affinity for hydrocarbon substrates [48]. This might explain the unstable emulsifications observed against diesel and sunflower oil. Our findings bear conformity with those reported by Rufino et al. [49] in the case of *C. lipolytica* cultivated using industrial residue as substrate. They reported that diesel and hexane were not emulsified effectively thereby, substantiating a differential activity of the emulsifier depending on its affinity for different hydrocarbon substrates. Similarly, stability of a biosurfactant under extreme environmental conditions are important parameters for consideration in usage under specific environmental conditions. The SL under study exhibited excellent stability over the evaluated range of pH, temperature, salinity indicating possibility of its usage in sectors like bioremediation and microbial enhanced oil recovery. Luna et al. [44] obtained similar results using the SL produced by *Candida sphaerica* UCP0995 from industrial wastes that showed stable ST reduction and emulsifying activity at different pH, temperature, and NaCl concentrations against motor oil.

The composition of the biosurfactant produced by *R. babjevae* YS3 was determined by TLC, FTIR and LC–MS analyses conducted in comparison with a standard lactonic sophorolipid (1,4′′-sophorolactone 6′,6′′-diacetate). The TLC analyses indicated that the test sophorolipid was composed of a mixture of acidic (AS) and lactonic sophorolipids (LS). Ribeiro et al. [35], reportedly obtained the R_*f*_ values of AS produced by *R. bogoriensis* in the comparable range of 0.18–0.41, eluted with the same solvent system as that used in the present study. The characteristic functional groups present in the column purified biosurfactant were determined by FTIR and compared with the standard SL. In addition to similar bands with the standard confirming the presence of lactonic SL, the presence of AS was confirmed by the two bands at 1445 and 3403 cm^−1^ typically associated with AS in literature [25]. Similar absorption bands were observed by Daverey and Pakshirajan [32] for a SL produced by *C. bombicola* grown on a fermentative medium containing sugarcane molasses, yeast extract, urea, and soybean oil. Electrospray ionization (ESI) of small molecules (~500 Da) with a single functional group capable of carrying electrical charge predominantly produce singly charged ions usually involving the addition of a proton to the analyte (M+H^+^), but adduction of cations (like M+Na^+^, M+K^+^) are also reported to occur if salts are present, when the ion source is in positive ion mode [50]. As such our analysis revealed both protonated as well as sodiated ions of the acidic (AS) and lactonic (LS) sophorolipids with variable fatty acid side chains (C_11_–C_18_). Chen et al. [51] reported a mixture of both AS (C_18_ Ac_2_, C_18:1_ Ac_2_, C_18:1_ Ac) and Ac_2_LS (C_18:2_, C_18:3_, C_18:2_, C_18:1_, C_18_, C_16_) on performing LC–MS analysis of the biosurfactant produced by *Wickerhamiella domercqiae*, indicating a similar heterogeneous composition and variability in the fatty acid portion. Our results also bear conformity with previous literature reporting a similar composition of homologues for the standard sophorolactone [29, 36].

Although, the largest application of biosurfactant is the oil industry, biosurfactants from many microorganisms have demonstrated antimicrobial properties and are currently being extracted and investigated to stem the incidence of antibiotic resistance plaguing the world today [48]. The antagonistic activity exhibited by biosurfactants might be attributed to the destabilization of cellular membrane causing cytoplasmic extrusions and eventually resulting in the rupture of cells [52]. In our study, the promising activity against *Colletotrichum gloeosporioides* indicates a potential application as food preservative against post-harvest decays of apple caused by this pathogen [53]. The results are of particular interest as previous literature describing appreciable antifungal activity of SLs are limited. Yoo et al. [54] reported a relatively lower, 8% inhibition of mycelial growth using 500 µg/ml SL against the *Phytophthora* sp. and *Pythium* sp. The same authors used 2000 µg/ml SL to obtain 42% reduction of damping-off disease in pot trials. The SL used in our study appear to be much more promising than that used by Yoo et al. [54]. However, no appreciable activity was observed against *Corynespora cassiicola* at the evaluated concentrations during our study. A similar variation in activity was also observed by Dengle-Pulate et al. [12] during their study involving a SL produced by *Candida bombicola* using glucose as the hydrophilic source and lauryl alcohol C_12–14_, as the hydrophobic source. They reportedly used a concentration of 50 µg/ml to obtain antagonistic activity against pathogenic yeast *Candida albicans* as against 6 and 1 μg/ml used to obtain complete inhibition of *Staphylococcus aureus* and *Bacillus subtilis* respectively. From their observations, it was evident that the activity of SL may vary against different pathogens which might explain the differential activity observed during our study.

## Conclusion

*Rhodotorula babjevae* YS3, isolated from soil collected from Assam, North East India produced sophorolipid with both acidic and lactonic forms. The sophorolipid showed good surface and emulsification activity along with an excellent stability over a wide range of pH, salinity, and temperature suggesting its possible use in environmental and large scale industrial applications. The potential antifungal activity exhibited by the sophorolipid also demonstrates prospects for its use in biomedical sector and as an alternative to synthetic agrochemicals. To our knowledge, this is the first report of the production of sophorolipid, or biosurfactant by *R. babjevae*.

## References

[1] Banat IM, Franzetti A, Gandolfi I, Bestetti G, Martinotti MG, Fracchia L, Smyth TJ, Marchant R (2010). Microbial biosurfactants production, applications and future potential. Appl Microbiol Biotechnol.

[2] Rahman PK, Gakpe E (2008). Production, characterisation and applications of biosurfactants-Review. Biotechnology.

[3] Desai JD, Banat IM (1997). Microbial production of surfactants and their commercial potential. Microbiol Mol Biol Rev.

[4] Cameotra SS, Makkar RS (2004). Recent applications of biosurfactants as biological and immunological molecules. Curr Opin Microbiol.

[5] Borah SN, Goswami D, Lahkar J, Sarma HK, Khan MR, Deka S (2015). Rhamnolipid produced by *Pseudomonas aeruginosa* SS14 causes complete suppression of wilt by *Fusarium oxysporum* f. sp. *pisi* in *Pisum sativum*. Biocontrol.

[6] Van Bogaert IN, Saerens K, De Muynck C, Develter D, Soetaert W, Vandamme EJ (2007). Microbial production and application of sophorolipids. Appl Microbiol Biotechnol.

[7] Konishi M, Imura T, Fukuoka T, Morita T, Kitamoto D (2007). A yeast glycolipid biosurfactant, mannosylerythritol lipid, shows high binding affinity towards lectins on a self-assembled monolayer system. Biotechnol Lett.

[8] Konishi M, Morita T, Fukuoka T, Imura T, Kakugawa K, Kitamoto D (2007). Production of different types of mannosylerythritol lipids as biosurfactants by the newly isolated yeast strains belonging to the genus Pseudozyma. Appl Microbiol Biotechnol.

[9] Cavalero DA, Cooper DG (2003). The effect of medium composition on the structure and physical state of sophorolipids produced by *Candida bombicola* ATCC 22214. J Biotechnol.

[10] Bluth MH, Kandil E, Mueller CM, Shah V, Lin Y-Y, Zhang H, Dresner L, Lempert L, Nowakowski M, Gross R (2006). Sophorolipids block lethal effects of septic shock in rats in a cecal ligation and puncture model of experimental sepsis. Crit Care Med.

[11] Haque F, Alfatah M, Ganesan K, Bhattacharyya MS (2016). Inhibitory effect of sophorolipid on *Candida albicans* biofilm formation and hyphal growth. Sci Rep.

[12] Dengle-Pulate V, Chandorkar P, Bhagwat S, Prabhune AA (2014). Antimicrobial and SEM studies of sophorolipids synthesized using lauryl alcohol. J Surfactants Deterg.

[13] Yurkov A, Kemler M, Begerow D (2012). Assessment of yeast diversity in soils under different management regimes. Fungal Ecol.

[14] Morikawa M, Daido H, Takao T, Murata S, Shimonishi Y, Imanaka T (1993). A new lipopeptide biosurfactant produced by *Arthrobacter* sp. strain MIS38. J Bacteriol.

[15] Youssef NH, Duncan KE, Nagle DP, Savage KN, Knapp RM, McInerney MJ (2004). Comparison of methods to detect biosurfactant production by diverse microorganisms. J Microbiol Methods.

[16] Cooper DG, Goldenberg BG (1987). Surface-active agents from two *Bacillus* species. Appl Environ Microbiol.

[17] Gardes M, Bruns TD (1993). ITS primers with enhanced specificity for basidiomycetes-application to the identification of mycorrhizae and rusts. Mol Ecol.

[18] White TJ, Bruns T, Lee S, Taylor J (1990). Amplification and direct sequencing of fungal ribosomal RNA genes for phylogenetics. PCR Protoc Guide Methods Appl.

[19] Hamby KA, Hernández A, Boundy-Mills K, Zalom FG (2012). Associations of yeasts with spotted-wing *Drosophila* (*Drosophila suzukii*; *Diptera*: *Drosophilidae*) in cherries and raspberries. Appl Environ Microbiol.

[20] Bourret TB, Grove GG, Vandemark GJ, Henick-Kling T, Glawe DA (2013). Diversity and molecular determination of wild yeasts in a central Washington State vineyard. N Am Fungi.

[21] Thompson JD, Higgins DG, Gibson TJ (1994). CLUSTAL W: improving the sensitivity of progressive multiple sequence alignment through sequence weighting, position-specific gap penalties and weight matrix choice. Nucleic Acids Res.

[22] Kumar S, Stecher G, Tamura K (2016). MEGA7: molecular evolutionary genetics analysis version 7.0 for bigger datasets. Mol Biol Evol.

[23] Garcıa-Ochoa F, Casas J (1999). Unstructured kinetic model for sophorolipid production by *Candida bombicola*. Enzyme Microb Technol.

[24] Luna J, Rufino R, Campos G, Sarubbo L (2012). Properties of the biosurfactant produced by *Candida sphaerica* cultivated in low-cost substrates. Chem Eng.

[25] Daverey A, Pakshirajan K (2010). Sophorolipids from *Candida bombicola* using mixed hydrophilic substrates: production, purification and characterization. Coll Surf B Biointerfaces.

[26] Bonilla M, Olivaro C, Corona M, Vazquez A, Soubes M (2005). Production and characterization of a new bioemulsifier from *Pseudomonas putida* ML2. J Appl Microbiol.

[27] Bordoloi N, Konwar B (2008). Microbial surfactant-enhanced mineral oil recovery under laboratory conditions. Coll Surf B Biointerfaces.

[28] Borah SN, Goswami D, Sarma HK, Cameotra SS, Deka S (2016). Rhamnolipid biosurfactant against *Fusarium verticillioides* to control stalk and ear rot disease of Maize. Front Microbiol.

[29] Samad A, Zhang J, Chen D, Liang Y (2015). Sophorolipid production from biomass hydrolysates. Appl Biochem Biotechnol.

[30] Washington J, Wood G, Murray P (1995). Antimicroibial susceptibility tests: dilution and disc diffusion methods. Manual of clinical microbiology.

[31] Arcuri SL, Pagnocca FC, da Paixão Melo WG, Nagamoto NS, Komura DL, Rodrigues A (2014). Yeasts found on an ephemeral reproductive caste of the leaf-cutting ant *Atta sexdens* rubropilosa. Antonie Van Leeuwenhoek.

[32] Daverey A, Pakshirajan K (2009). Production, characterization, and properties of sophorolipids from the yeast *Candida bombicola* using a low-cost fermentative medium. Appl Biochem Biotechnol.

[33] Asmer H-J, Lang S, Wagner F, Wray V (1988). Microbial production, structure elucidation and bioconversion of sophorose lipids. J Am Oil Chem Soc.

[34] Mousavi F, Beheshti-Maal K, Massah A (2015). Production of sophorolipid from an identified current yeast, *Lachancea thermotolerans* BBMCZ7FA20. Isol Honey Bee Curr Microbiol.

[35] Ribeiro IA, Bronze MR, Castro MF, Ribeiro MH (2012). Optimization and correlation of HPLC-ELSD and HPLC–MS/MS methods for identification and characterization of sophorolipids. J Chromatogr B.

[36] Ashby RD, Zerkowski JA, Solaiman DK, Liu LS (2011). Biopolymer scaffolds for use in delivering antimicrobial sophorolipids to the acne-causing bacterium *Propionibacterium acnes*. New Biotechnol.

[37] Jain D, Collins-Thompson D, Lee H, Trevors J (1991). A drop-collapsing test for screening surfactant-producing microorganisms. J Microbiol Methods.

[38] Sobrinho HB, Rufino RD, Luna JM, Salgueiro AA, Campos-Takaki GM, Leite LF, Sarubbo LA (2008). Utilization of two agroindustrial by-products for the production of a surfactant by *Candida sphaerica* UCP0995. Process Biochem.

[39] Banat IM (1995). Biosurfactants production and possible uses in microbial enhanced oil recovery and oil pollution remediation: a review. Bioresour Technol.

[40] Desai JD, Desai AJ, Kosaric N (1993). Production of biosurfactants. Biosurfactants: production properties applications.

[41] Diniz Rufino R, Moura de Luna J, de Campos Takaki GM, Asfora Sarubbo L (2014). Characterization and properties of the biosurfactant produced by *Candida lipolytica* UCP 0988. Electron J Biotechnol.

[42] Accorsini FR, Mutton MJR, Lemos EGM, Benincasa M (2012). Biosurfactants production by yeasts using soybean oil and glycerol as low cost substrate. Braz J Microbiol.

[43] de Souza Sobrinho BH, de Luna JM, Rufino RD, Figueiredo Porto AL, Sarubbo LA (2013). Assessment of toxicity of a biosurfactant from *Candida sphaerica* UCP 0995 cultivated with industrial residues in a bioreactor. Electron J Biotechnol.

[44] Luna JM, Rufino RD, Sarubbo LA, Campos-Takaki GM (2013). Characterisation, surface properties and biological activity of a biosurfactant produced from industrial waste by *Candida sphaerica* UCP0995 for application in the petroleum industry. Coll Surf B Biointerfaces.

[45] Haba E, Abalos A, Jauregui O, Espuny M, Manresa A (2003). Use of liquid chromatography-mass spectroscopy for studying the composition and properties of rhamnolipids produced by different strains of *Pseudomonas aeruginosa*. J Surfactants Deterg.

[46] Pacwa-Płociniczak M, Płaza GA, Piotrowska-Seget Z, Cameotra SS (2011). Environmental applications of biosurfactants: recent advances. Int J Mol Sci.

[47] Banat IM, Makkar RS, Cameotra S (2000). Potential commercial applications of microbial surfactants. Appl Microbiol Biotechnol.

[48] Ilori M, Amobi C, Odocha A (2005). Factors affecting biosurfactant production by oil degrading *Aeromonas* spp. isolated from a tropical environment. Chemosphere.

[49] Rufino R, Sarubbo L, Campos-Takaki G (2007). Enhancement of stability of biosurfactant produced by *Candida lipolytica* using industrial residue as substrate. World J Microbiol Biotechnol.

[50] Pitt JJ (2009). Principles and applications of liquid chromatography–mass spectrometry in clinical biochemistry. Clin Biochem Rev.

[51] Chen J, Zhang H, Liu Y, Fu S, Liu X (2014). Metal ions can affect the composition and production of sophorolipids by *Wickerhamiella domercqiae* Y2A CGMCC 3798. Eur J Lipid Sci Technol.

[52] Hirata Y, Ryu M, Oda Y, Igarashi K, Nagatsuka A, Furuta T, Sugiura M (2009). Novel characteristics of sophorolipids, yeast glycolipid biosurfactants, as biodegradable low-foaming surfactants. J Biosci Bioeng.

[53] Sharma M, Kulshrestha S (2015). *Colletotrichum gloeosporioides*: an anthracnose causing pathogen of fruits and vegetables. Biosci Biotechnol Res Asia.

[54] Yoo DS, Lee BS, Kim EK (2005). Characteristics of microbial biosurfactant as an antifungal agent against plant pathogenic fungus. J Microbiol Biotechnol.

